# High-Touch, High-Risk: An Exploratory Microbiome Analysis of Hospital Wheelchairs

**DOI:** 10.3390/pathogens15070693

**Published:** 2026-06-30

**Authors:** Luca Dalle Carbonare, Anna Vareschi, Kevin Dervishi, Michela Deiana, Elena Locatelli, Arianna Minoia, Francesca Cristiana Piritore, Alessandra Ruggiero, Ilda Czobor Barbu, Donato Zipeto, Chiara Piubelli, Maria Teresa Valenti

**Affiliations:** 1 Department of Engineering for the Innovation Medicine, University of Verona, 37100 Verona, Italy; luca.dallecarbonare@univr.it (L.D.C.); anna.vareschi@univ.it (A.V.); arianna.minoia@univr.it (A.M.); 2 Department of Neurosciences, Biomedicine and Movement Sciences, University of Verona, 37100 Verona, Italy; kevin.dervishi@univr.it (K.D.); francescacristiana.piritore@univr.it (F.C.P.); alessandra.ruggiero@univr.it (A.R.); donato.zipeto@univr.it (D.Z.); 3 Department of Infectious, Tropical Diseases and Microbiology, IRCCS Sacro Cuore Don Calabria Hospital, 37024 Negrar di Valpolicella, Italy; michela.deiana@sacrocuore.it (M.D.); elena.locatelli@sacrocuore.it (E.L.); chiara.piubelli@sacrocuore.it (C.P.); 4 Department of Botany and Microbiology, Faculty of Biology, University of Bucharest, 030018 Bucharest, Romania; ilda.barbu@bio.unibuc.ro

**Keywords:** hospital wheelchairs, microbial contamination, high-touch surfaces, next-generation sequencing (NGS), infection control, protective barrier

## Abstract

In this exploratory pilot study, quantitative analyses were performed on seven leather wheelchairs and the protective barrier was evaluated on three leather wheelchairs, while shotgun metagenomic sequencing (Illumina and Oxford Nanopore) was conducted on pooled samples obtained from seven leather and three fabric wheelchairs to characterize microbial DNA recovered from wheelchair surfaces under routine clinical conditions. Microbial DNA and biomass were detected on all sampled surfaces, with median DNA concentrations of approximately 0.015 ng/µL, median cell counts of approximately 4.8 × 10^5^ cells/mL, and median OD600 values of approximately 0.038, although variability among wheelchairs was observed. NGS analysis revealed heterogeneous microbial communities composed mainly of taxa associated with human skin microbiota and environmental sources. Opportunistic taxa including *Escherichia coli*, *Staphylococcus haemolyticus*, *Achromobacter xylosoxidans*, and *Clostridioides difficile* DNA were detected. Differences in microbial composition were observed between the pooled fabric and leather samples, with fabric samples characterized by the dominance of specific taxa and leather samples exhibiting a more heterogeneous microbial profile. In addition, median DNA concentration, cell counts, and OD600 values were reduced by approximately 98–100% on the protective barrier compared with uncovered wheelchair surfaces, with statistically significant differences between conditions. Overall, these findings suggest that hospital wheelchairs may harbor measurable levels of microbial biomass and microbial DNA despite routine sanitation procedures. Lower contamination levels were observed on the protective barrier under the conditions tested. Due to the exploratory nature of the study, the small sample size, and the use of pooled samples for metagenomic analyses, these observations should be interpreted with caution and require confirmation in larger studies.

## 1. Introduction

Healthcare-associated infections (HAIs) remain a persistent challenge for healthcare systems, contributing substantially to patient morbidity, mortality and increased healthcare costs worldwide [1,2]. Among the various transmission pathways, indirect contact via contaminated surfaces is increasingly recognized as an important mechanism for the spread of microorganisms in clinical environments [3,4]. High-contact objects, which patients, healthcare workers and visitors frequently touch, can act as reservoirs that facilitate microbial persistence and transmission [5]. Environmental reservoirs are increasingly recognized as important components of healthcare-associated infection ecology, particularly in the context of emerging antimicrobial resistance. Although environmental contamination alone does not demonstrate transmission, shared high-touch surfaces may contribute to the persistence and dissemination of opportunistic microorganisms, including taxa frequently associated with healthcare settings and antimicrobial resistance.

Infection prevention strategies have traditionally focused on well-defined environmental surfaces such as bed rails, medical devices and workstations [6]. In contrast, shared mobility aids have received comparatively limited attention, despite their extensive use across different clinical settings [7,8]. Hospital wheelchairs represent a particularly critical interface, as they are continuously transferred between users and departments, often under conditions of variable cleaning schedules and heterogeneous sanitation practices [7,8]. Their structural composition, which includes both non-porous and porous materials, may further promote microbial retention.

Most current knowledge about surface contamination in healthcare settings comes from culture-based studies, which capture only a fraction of microbial diversity and may underestimate the complexity of contamination [9,10,11]. The application of shotgun metagenomics sequencing has significantly expanded the ability to characterize environmental microbiota, revealing diversified microbial communities on hospital surfaces [12,13,14]. However, these approaches remain poorly applied for the characterization of microbiota on shared mobility devices. This is primarily due to the intrinsic analytical complexity of processing samples from diverse materials and the significant costs associated with shotgun sequencing.

Despite these barriers, wheelchairs warrant specific investigation as they may represent a unique reservoir of microbial contamination. Furthermore, the role of material properties in shaping microbial community composition remains incompletely understood, particularly under routine clinical conditions. Differences between porous and non-porous surfaces may influence both microbial load and diversity, with potential impact on cleaning protocols and infection control. In addition, disposable protective barriers may represent a simple and practical strategy to reduce contact between users and shared wheelchair surfaces, although their effectiveness under routine clinical conditions remains poorly characterized.

Here, we investigate microbiological contamination on hospital wheelchairs in a routine clinical setting in Verona, Italy, using an integrated approach that combines quantitative assessment of total recovered material with hybrid shotgun metagenomic sequencing. To increase the sensitivity and robustness of taxonomic detection, two complementary sequencing technologies were used (Illumina and Oxford Nanopore Technologies), combining the high accuracy of short reads with the improved taxonomic resolution provided by long reads.

This approach provides a comprehensive framework for understanding the hygienic relevance of shared mobility devices and supports the development of targeted infection control strategies.

## 2. Materials and Methods

### 2.1. Study Design and Setting

The study was conducted at the Azienda Ospedaliera Universitaria Integrata (AOUI) of Verona, Italy, with the aim of assessing biomass contamination on hospital wheelchairs using an integrated approach that combined quantitative and sequencing-based analyses. Shotgun metagenomics sequencing analysis was performed at IRCCS Sacro Cuore Don Calabria Hospital. Different swab systems were used based on prior optimization results, with each method applied consistently within its respective workflow.

The study included three related but distinct experimental components. Quantitative contamination analyses (DNA concentration, cell counts, and OD600) were performed on seven leather wheelchairs. The barrier experiment was conducted on three leather wheelchairs to assess contamination detected on a disposable protective cover under routine transport conditions. Metagenomic analyses were performed separately on pooled samples obtained from seven leather wheelchairs and three fabric wheelchairs to characterize microbial community composition. These sample sets were used for different analytical purposes and should not be interpreted as a single uniform cohort. No formal sample size calculation was performed because this was an exploratory pilot study.

### 2.2. Quantitative Assessment of Total Recovered Material

A total of seven hospital wheelchairs made of leather from different hospital departments (Internal Medicine, Emergency Department, and outpatient clinic areas) were included in the study. Sampling was performed on high-contact surfaces, specifically handles, armrests, and seats, which are frequently touched during routine use. For handles, the entire accessible surface was swabbed. For armrests and seats, comparable high-contact surface areas were sampled to ensure consistency among wheelchairs. Surface material was collected using environmental sampling swabs (Neogen^®^ Quick Swab; Neogen Corporation, Lansing, MI, USA). After collection, each swab was immersed in 10 mL of sterile phosphate-buffered saline (PBS) (Thermo Fisher Scientific Inc., Waltham, MA, USA) and vortexed for 1 min to ensure efficient recovery and homogenization of the microbial content.

#### 2.2.1. Quantitative Evaluation of Total Recovered Material

The material recovered from the sampled surfaces, including biological (microbial and non-microbial) and non-biological components (e.g., textile fibers and particulate matter), was evaluated using complementary quantitative approaches. Optical density at 600 nm (OD600) was measured using a Eppendorf Biophotometer (Eppendorf AG, Hamburg, Germany) to evaluate the suspension turbidity as we previously described [15]. Cell counts were further determined using a Bürker counting chamber. Only morphologically identifiable cellular structures were counted. The method was intended to provide an estimate of total cellular material recovered from the sampled surfaces. Samples were observed under a brightfield microscope at 40× magnification, and cells were manually enumerated within defined grid areas. 

#### 2.2.2. DNA Extraction and Quantification for Biological Load Evaluation

Total DNA was extracted from the samples using a column-based protocol (Cat. No./ID: 51104, QIAGEN, Hilden, Germany), following the manufacturer’s instructions. DNA concentration was assessed using a Qubit fluorometer (Thermo Fisher Scientific Inc., Waltham, MA, USA) with a high-sensitivity assay, and values were expressed in ng/µL.

#### 2.2.3. Evaluation of Protective Barrier Under Routine Hospital Conditions

The effectiveness of a fabric/nonwoven protective barrier (SAFE-HUG PRO Wheelchair Cover; EASYLINKED S.r.l., Milan, Italy) was evaluated under controlled experimental conditions. Three hospital leather wheelchairs were included in the analysis. For each wheelchair, three high-contact sampling sites (handles, armrests, and seats) were sampled separately. Measurements were obtained from uncovered wheelchair surfaces (uncovered condition) and from the protective barrier after its application (covered condition), yielding a total of nine site-level observations for each condition. To avoid bias due to prior sampling, measurements were obtained from adjacent, non-overlapping, comparable surface areas. The protective cover is intended as a single-use barrier during patient transport. Therefore, contamination was assessed after approximately 30 min of routine use, corresponding to the typical duration of a patient transport episode within the hospital. Sampling analyses were performed as described above, allowing direct comparison of contamination levels between conditions and assessment of the barrier’s potential to reduce accumulation on high-contact surfaces.

#### 2.2.4. Statistical Analysis

Statistical analysis was performed to compare biomass contamination between covered and uncovered wheelchairs. For the barrier experiment, three sampling locations (handles, armrests, and seats) were analyzed on each of three wheelchairs, yielding nine site-level observations per condition. Differences in total DNA concentration, cell counts, and optical density (OD600) between conditions were evaluated using the non-parametric Mann–Whitney test. Data are presented as median and interquartile range (IQR) in box-and-whisker plots. A *p*-value < 0.05 was considered statistically significant. Statistical analyses were performed using GraphPad Prism version 9 (GraphPad Software, San Diego, CA, USA).

### 2.3. Metagenomic Analyses

#### 2.3.1. Optimization of Sampling and Extraction Procedures

A pilot study was conducted to identify the most effective sampling and DNA extraction strategy for NGS analysis. Different swab types, including e-swabs (Copan, Brescia, Italy), Quick swabs (Neogen, Lansing, MI, USA) and Enviro swabs (Neogen, Lansing, MI, USA) were tested on both fabric and leather wheelchair surfaces. In parallel, two DNA extraction kits (Monarch Genomic DNA Purification Kit, NEB, Ipswich, MA, USA; and Qiagen blood and tissue kit, (QIAGEN, Hilden, Germany) were compared on the basis of DNA yield and consistency. The pilot study indicated that e-swabs provided the most reliable DNA recovery, while the Monarch extraction kit yielded higher DNA concentrations and improved reproducibility. These conditions were therefore selected for subsequent sequencing analyses of wheelchairs made of fabric (*n* = 3) and leather (*n* = 7).

#### 2.3.2. NGS Sampling and Sequencing Workflow

Following optimization, bulk sampling was performed to characterize microbial communities associated with different surface materials. Wheelchairs were grouped according to material type, and pooled samples were collected separately from fabric and leather surfaces using e-swabs.

DNA extracted using the optimized protocol was subjected to whole-genome amplification (WGA) before library preparation. A negative extraction control was included in the workflow by processing a blank e-swab sample. The control yielded no quantifiable DNA after extraction. Following whole genome amplification, only a trace amount of DNA (~0.01 ng/μL) was detected, and no amplification products were visible upon TapeStation analysis. Because the DNA yield was below the threshold required for library preparation and no detectable DNA profile was observed, the negative control was not carried forward to sequencing. Shotgun metagenomic libraries were prepared from 100 ng of input DNA using the Illumina DNA Prep Kit, according to manufacturer instructions. Libraries were sequenced on a NextSeq1000 platform (Illumina, San Diego, CA, USA) to generate high-depth short-read data in a 2 × 150 bp configuration. In parallel, long-read sequencing was performed starting from 1 µg of input DNA per sample, using Oxford Nanopore Technologies (ONT, Oxford, UK). Libraries were prepared using the Native Barcoding Kit and sequenced on a MinION Mk1D platform (ONT) (Oxford Nanopore Technologies, Oxford, UK). Library quality control included DNA quantification and assessment of fragment size distribution.

#### 2.3.3. Bioinformatic Analysis

Data derived from the two different sequencing approaches were initially analyzed separately (i.e., fabric Illumina, fabric ONT, leather Illumina and leather ONT). Sequencing data were processed using CZ ID [16], a cloud-based bioinformatics platform for metagenomic analysis and taxonomic classification. 

Specific filtering thresholds were applied according to the sequencing platform. Illumina data were filtered according to the number of reads per million (NT rpm ≥ 100), percentage of identity (≥95%), the length of the alignment (≥100 bp), and non-redundant protein database reads per million (NR rpm ≥ 10). Similarly, the ONT data were also filtered using adjusted thresholds to account for platform-specific characteristics, including nucleotide read count (NT reads ≥ 10), percentage of identity (≥92%), nucleotide alignment length (≥100 bp), and non-redundant protein bases per million (NR bpm ≥ 10).

Final taxonomic profiles were obtained by retaining taxa that passed quality filters in at least one of the two platforms. The average (Log10) relative abundance was reported for the detected taxa.

## 3. Results

### 3.1. Quantitative Assessment of Total Recovered Material

**Figure 1:**
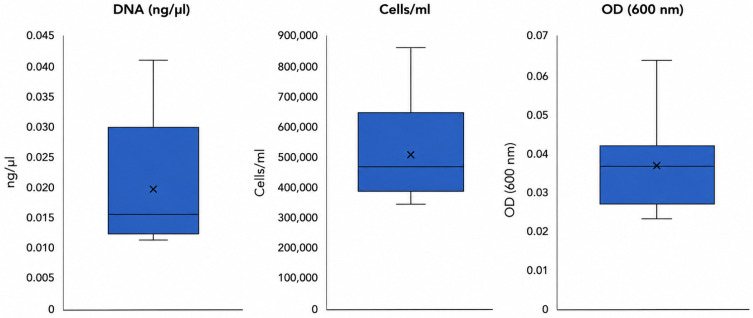
Quantitative assessment of contamination on hospital wheelchairs. Total DNA concentration, cell counts and optical density (OD600), are reported for samples collected from high-contact surfaces (handles, armrests, and seats) of seven hospital wheelchairs. Boxes represent the interquartile range, the horizontal line indicates the median, whiskers represent the minimum and maximum values, and × indicates the mean.

Swab analysis of hospital wheelchairs (*n* = 7) revealed detectable DNA in all sampled surfaces, consistent with the ubiquitous presence of environmental and human-associated microorganisms on frequently handled objects. Quantitative measurements showed detectable cell loads, with variability among wheelchairs (Figure 1). Optical density (OD600) values were consistently above baseline levels in all samples (Figure 1), indicating detectable surface-associated biomass. Similarly, cell counts confirmed the presence of cellular populations, although with heterogeneous distribution across the different wheelchairs analyzed (Figure 1). Despite this variability, microbial biomass was consistently detectable across all sampled wheelchairs, consistent with environments subject to routine sanitation practices. 

### 3.2. Effect of Protective Barrier on Contamination

**Figure 2:**
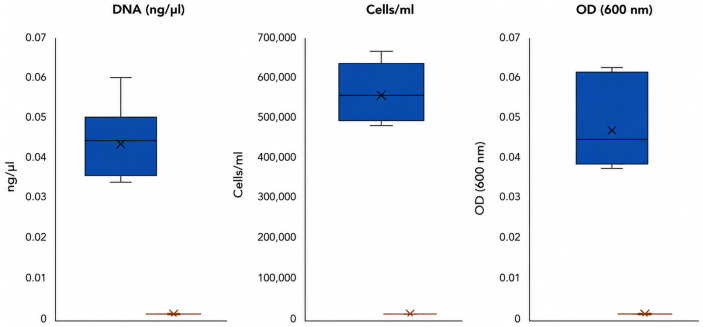
Quantitative assessment of contamination levels on wheelchair surface measured before (uncovered) and after (covered) application of the protective barrier. Comparison of total DNA concentration (ng/µL), cell counts (cells/mL), and optical density (OD600) between uncovered (blue) and covered (orange) wheelchairs under routine clinical conditions. Data are shown as box-and-whisker plots representing the median and interquartile range (IQR) based on nine site-level observations per condition. Statistical significance was assessed using the Mann–Whitney test.

To evaluate the effectiveness of a fabric/nonwoven protective barrier applied to hospital wheelchairs under routine clinical use, contamination levels were compared between uncovered conditions and those measured on the protective barrier after 30 min of application. Analysis of total DNA concentration showed a clear difference between the two groups. Uncovered wheelchairs displayed measurable and variable DNA levels, whereas covered wheelchairs exhibited markedly reduced concentrations, often approaching detection limits (Figure 2). Cell count analysis showed lower biomass levels on the protective barrier compared with uncovered wheelchair surfaces, suggesting lower biomass levels under the conditions tested. Uncovered wheelchairs showed detectable cellular populations with variability across samples, whereas covered wheelchairs consistently presented cell counts close to zero (Figure 2). Optical density measurements (OD600) further supported these findings. Higher OD values were observed in uncovered wheelchairs, indicating detectable biomass, whereas values in covered wheelchairs remained close to baseline, suggesting minimal biomass levels (Figure 2). The differences observed between covered and uncovered wheelchairs were statistically significant for total DNA concentration (Mann–Whitney *p* = 0.000165), cell counts (*p* = 0.000041), and OD (*p* = 0.000041).

### 3.3. Metagenomic Sequencing Yields and Reads Quality Control

Illumina short reads sequencing produced 30M reads (average). ONT sequencing generated 84K reads (average), with mean lengths ranging from approximately 3.3 kb to 6.7 kb. Table 1 and Table 2 summarized sequencing details of the two sequencing platforms across the tested samples. 

### 3.4. Taxonomic Profiling of Microbial Communities

Taxonomic analysis revealed diverse microbial communities in both fabric and leather wheelchair samples. Across all datasets, bacterial taxa represented the dominant component of the microbiome, accompanied by viral and eukaryotic sequences. Several taxa commonly associated with human skin and environmental sources were consistently detected, including *Staphylococcus* spp., *Cutibacterium acnes*, and *Pseudomonas* spp. 

Leather-associated samples showed a heterogeneous microbial composition, with the presence of environmental and commensal bacteria such as *Desemzia incerta*, *Anoxybacillus caldiproteolyticus*, and *Rhodococcus* spp., together with taxa of potential clinical relevance, including *Staphylococcus haemolyticus*, *Escherichia coli*, and *Clostridioides difficile* (toxins not detected) (Table 3). Fabric-associated samples exhibited a distinct microbial profile characterized by higher relative abundance of Gram-negative bacteria, including *E. coli*, *Achromobacter xylosoxidans*, and members of the *Pseudomonas fluorescens* group (Table 4). These taxa were consistently identified across both sequencing platforms, supporting the robustness of the observed patterns.

### 3.5. Comparison Between Sequencing Platforms

Concordance between Illumina and ONT dataset/platform was assessed by comparing microbial taxonomic profiles. Leather swab samples revealed the presence of 18 taxa (14 bacteria, 1 eukaryote, and 3 viruses) and 8 taxa (bacteria) for Illumina and ONT platform, respectively, while fabric swab samples identified 7 bacterial species on Illumina platform and 4 bacterial species on ONT platform.

Both platforms showed an overall consistency in dominant species detection across samples. Illumina provided a higher read depth, while Oxford Nanopore Technologies contributed longer reads improving taxonomic assignment, although differences in sensitivity and taxonomic resolution were observed.

### 3.6. Relative Abundance and Community Structure

**Figure 3:**
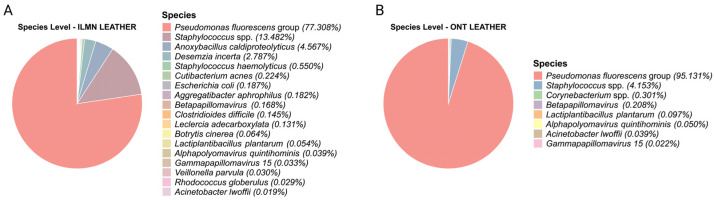
Relative abundance of microbial taxa in leather wheelchair samples. (**A**) Illumina-based metagenomic profiling showing the relative abundance of microbial species detected in leather surfaces. (**B**) Oxford Nanopore Technologies (ONT) profiling of the same sample type, illustrating species-level composition based on long-read sequencing.

**Figure 4:**
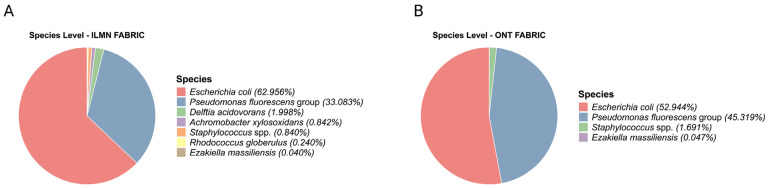
Relative abundance of microbial taxa in fabric wheelchair samples. (**A**) Illumina sequencing-based profiling of microbial communities at species level. (**B**) Oxford Nanopore Technologies (ONT) sequencing-based profiling of the same sample type.

Relative abundance analysis revealed that a limited number of taxa accounted for the majority of reads within each sample. Pie chart representations showed clear dominance patterns, with *Pseudomonas* spp., *E. coli*, and *Staphylococcus* spp. among the most represented taxa across datasets (Figure 3 and Figure 4). When sequencing data from both platforms were combined and log-transformed normalization was applied, comparative analysis between materials highlighted differences in microbial composition. 

**Figure 5:**
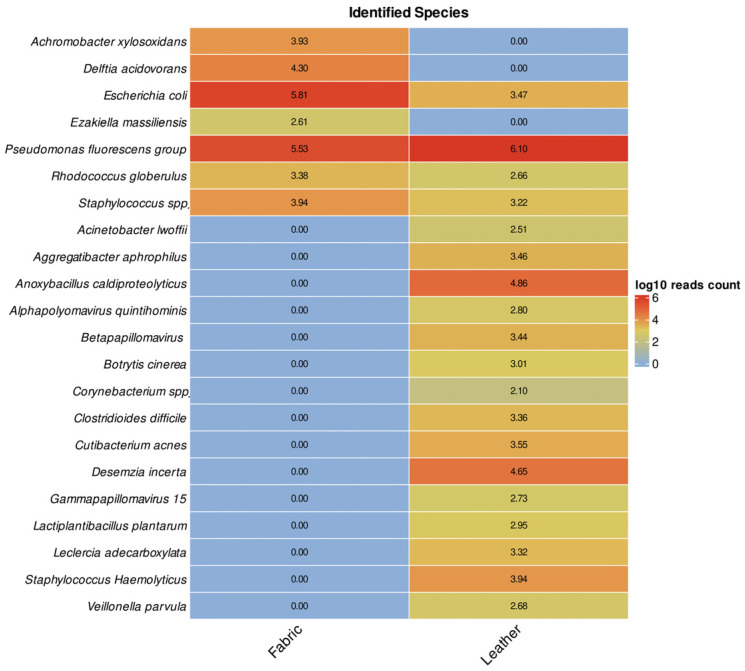
Heatmap of log10 transformed read counts from leather and fabric samples.

Heatmap visualization demonstrated differences between the pooled fabric and leather samples (Figure 5). However, due to sample pooling and the absence of biological replication, these observations should be interpreted as descriptive and exploratory.

## 4. Discussion

This study provides an integrated evaluation of the total biological load recovered from the sampled surfaces on hospital wheelchairs, complemented by sequencing-based characterization of microbial communities. Moreover, a protective barrier under routine clinical conditions has been assessed.

The quantitative results showed that “biomass” contaminant (which can include microbial cells, human-derived material, and environmental debris or particulate such as pollen) was consistently detectable on all sampled wheelchairs, although at moderate levels and with variability among samples. These findings are consistent with previous evidence indicating that high-contact surfaces in healthcare settings can act as reservoirs of microorganisms despite routine sanitation procedures [5,17,18,19]. 

Shared patient equipment, including wheelchairs, occupies a particular position within healthcare environments because it is used by multiple individuals and frequently transferred across wards and clinical areas. Although direct transmission was not assessed in the present study, contamination of high-touch shared equipment may contribute to indirect microbial transfer in healthcare settings. The epidemiological relevance of contaminated surfaces depends not only on the presence of microbial material but also on factors such as microbial persistence, environmental survival, cleaning effectiveness, and the probability of transfer to patients or healthcare workers, none of which were directly evaluated here. Previous studies have reported microbial contamination on hospital wheelchairs at levels comparable to those observed on other high-touch surfaces, including bed rails, bedside tables, infusion pumps, and shared medical equipment. In this context, the present study extends previous observations by combining quantitative biomass assessment with shotgun metagenomic characterization of wheelchair-associated microbial communities and by providing an exploratory evaluation of a disposable protective barrier under routine hospital conditions. Although antimicrobial resistance was not directly investigated, the identification of taxa previously associated with healthcare-associated infections highlights the importance of understanding contamination patterns on shared patient equipment. Environmental surveillance approaches may contribute to infection prevention strategies and provide complementary information for future investigations addressing antimicrobial resistance in healthcare environments. Importantly, the use of a fabric/nonwoven protective barrier resulted in a consistent reduction in biomass contamination across all quantitative parameters, including DNA concentration, cell counts, and optical density. These findings suggest that the barrier may act as a physical interface that limits direct contact between users and wheelchair surfaces, and lower contamination levels were observed on the barrier under the conditions tested. The protective barrier evaluated in this study should not be considered a substitute for established infection prevention and control measures, including routine cleaning and disinfection protocols, environmental monitoring programs, antimicrobial surface technologies, and ultraviolet disinfection systems. Rather, its potential value lies in serving as a simple, low-cost complementary intervention that may reduce contamination of shared high-touch equipment during routine patient transport. The relative effectiveness, feasibility, and cost–benefit profile of such barriers compared with other infection control strategies warrant further investigation.

Sequencing-based analyses revealed heterogeneous microbial communities across both fabric and leather surfaces, composed primarily of taxa associated with human skin and environmental sources. Several bacterial taxa, including *Pseudomonas* spp., *Escherichia coli*, and *Staphylococcus* spp., were consistently detected across both materials, suggesting the presence of a shared core microbiome likely reflecting repeated human contact and environmental exposure.

Differences in microbial composition and relative abundance were observed between materials. Fabric surfaces were characterized by the dominance of a limited number of taxa, including *Escherichia coli* and members of the *Pseudomonas fluorescens* group. In contrast, leather surfaces exhibited a more heterogeneous microbial composition, with the presence of multiple environmental and commensal taxa such as *Anoxybacillus caldiproteolyticus* and *Desemzia incerta*. However, because metagenomic analyses were performed on pooled samples without biological replication, these observations should be interpreted as descriptive and exploratory rather than evidence of a material-driven effect on microbial community structure. These differences may be influenced not only by microbial retention but also by sampling efficiency. The porous structure of fabric surfaces may retain biological material, potentially limiting recovery during swabbing, whereas smoother, non-porous surfaces such as leather may allow more consistent microbial collection. However, the number of sampled fabric wheelchairs was lower compared to leather ones. Notwithstanding that the amount of sequenced DNA was equal among the two groups, pooling differences could have influenced the abundance of detected taxa. 

From a clinical perspective, the taxa identified should be interpreted with caution and within a framework that accounts for pathogenic potential, host context, and the inherent limitations of DNA-based detection methods. Metagenomic sequencing identifies microbial genetic material but does not provide direct evidence of organism viability, infectivity, transmission potential, or infection risk. Therefore, the detection of potentially pathogenic taxa should not be interpreted as evidence of active contamination capable of causing infection. The identified species in fact generally constitute the main part of the human skin microbiota, but can be implicated in opportunistic infections in immunocompromised subjects, particularly in hospitalized patients. For instance, *E. coli* and coagulase-negative staphylococci, particularly *Staphylococcus haemolyticus*, are well-recognized opportunistic pathogens associated with infections across a broad range of clinical contexts, including bloodstream infections, UTIs and device-related complications [20,21,22,23,24,25]. *S. haemolyticus* has been specifically described as a cause of severe infections such as meningitis, endocarditis, prosthetic joint infections and bacteremia and is also known to cause septicemia, peritonitis, otitis media and diabetic foot ulcer infections [24]. Moreover, *S. haemolyticus* poses a substantial challenge in the battle against nosocomial infections due to its notable resistance to antimicrobials and proficient biofilm-forming capabilities [26,27]. Although *Clostridioides difficile* was detected in leather samples, no toxin-encoding genes were identified. This finding reduces the immediate clinical significance of the observation, since toxigenic strains are the principal cause of disease. However, given the limited sequencing depth and low input DNA, the absence of the toxins encoding genes may reflect insufficient coverage of low-abundance genomic regions rather than true absence. Nevertheless, *C. difficile* remains particularly relevant in immunocompromised populations, in whom colonization, infection, and recurrence are more frequent than in the general population, and its detection on shared mobility devices warrants continued surveillance, particularly in high-risk clinical units. *Achromobacter xylosoxidans* and *Delftia acidovorans*, although uncommon, have also been associated with bloodstream and device-related infections, often in patients with underlying conditions or impaired immunity. Among Gram-negative bacteria, *A. xylosoxidans* has been reported as an opportunistic pathogen in healthcare settings, particularly in immunocompromised patients. Clinical series have described its involvement in nosocomial bacteremia, frequently associated with underlying conditions such as malignancy and immunosuppression, as well as with device-related infections. Although relatively uncommon, infections caused by *A. xylosoxidans* may be associated with significant morbidity and, in severe cases, high mortality rates [28]. *D. acidovorans* is primarily an environmental organism with limited pathogenic potential, although sporadic infections have been reported, mainly in hospitalized or immunocompromised patients. Its detection in this context is therefore most likely related to environmental exposure rather than direct clinical relevance [29,30]. Similarly, members of the *Pseudomonas fluorescens* group are primarily considered environmental and of low pathogenic potential in humans; however, sporadic opportunistic infections have been reported, typically associated with contaminated medical equipment, warranting cautious interpretation in vulnerable hosts [31,32]. *Desemzia incerta*, a Gram-positive environmental bacterium with no established role in human disease, should be interpreted as part of the background microbiota rather than as evidence of clinically relevant contamination.

Overall, the detection of microbial DNA should not be equated with active infection risk. The clinical significance of the detected taxa depends on multiple factors, including microbial viability, inoculum, host susceptibility, antimicrobial exposure, and the presence of breaches in skin or mucosal barriers. 

This study has several limitations. First, the sample size was relatively small, particularly for the barrier experiment, which included only three wheelchairs. No formal sample size calculation was performed, as the study was exploratory in nature. Therefore, the findings should be interpreted with caution. In addition, the barrier experiment included multiple site-level observations (handles, armrests, and seats) obtained from the same wheelchair. Although these measurements were analyzed separately to capture variability across high-contact surfaces, they may not be fully independent. Therefore, the possibility of pseudoreplication cannot be excluded, and the statistical significance of the barrier-related findings should be interpreted with caution. Future studies involving substantially larger numbers of wheelchairs and independent biological replicates will be necessary to validate the contamination patterns observed in the present pilot study and to determine their generalizability across different healthcare settings. Second, metagenomic analyses were performed on pooled samples grouped by material type. Consequently, the sequencing results provide a descriptive overview of microbial communities associated with fabric and leather surfaces but do not allow assessment of inter-wheelchair variability. In addition, whole-genome amplification (WGA) was performed prior to library preparation because of the limited amount of recovered DNA. Although this approach enabled metagenomic sequencing, amplification bias may have influenced the relative abundance of detected taxa and should be considered when interpreting sequencing results. In addition, contamination on routinely used uncovered wheelchair surfaces reflected accumulation over a longer and uncontrolled period, whereas contamination on the protective barrier was assessed after approximately 30 min of use, corresponding to its intended single-use application during patient transport. Consequently, differences between conditions may partially reflect differences in cumulative exposure time. Information regarding the time elapsed since the last routine cleaning or disinfection procedure was not available for the sampled wheelchairs. Consequently, this factor could not be controlled and may have contributed to variability in contamination levels among samples. Furthermore, the study did not include culture-based analyses or other methods capable of distinguishing specific viable microorganisms. Future studies incorporating culture-based approaches or similar viability assays will be important to better define the clinical relevance of these findings. Although stringent bioinformatic filtering criteria were applied, the possibility of low-level background contamination cannot be completely excluded. Finally, the study did not include direct assessment of microbial transmission, nor did it include formal diversity indices for quantitative comparison of microbial communities. Despite these limitations, the combined use of quantitative and sequencing-based approaches provides a comprehensive overview of contamination patterns on hospital wheelchairs. The inclusion of a real-world evaluation of a protective barrier represents a practical contribution, suggesting that simple interventions may effectively reduce microbial contamination of shared medical devices in routine clinical settings.

## 5. Conclusions

Within the limitations of this exploratory study, hospital wheelchairs were found to harbor measurable levels of microbial biomass despite routine sanitation procedures. The combined quantitative and metagenomic approach revealed moderate but ubiquitous contamination, primarily composed of skin-associated and environmental taxa, with the occasional detection of opportunistic pathogens. Differences in microbial composition were observed between pooled fabric and leather samples; however, due to the absence of biological replication in the metagenomic analysis, these findings should be considered descriptive and require confirmation in larger studies using individual sample sequencing. Lower contamination levels were observed on the protective barrier under the conditions tested; however, larger studies are required to confirm these observations. Overall, these findings suggest that shared mobility devices warrant consideration within hospital hygiene and infection prevention programs. However, given the exploratory nature of the study and the absence of transmission assessments, further investigations are needed to determine the clinical significance of the observed contamination patterns and the potential role of preventive strategies in reducing microbial burden on shared equipment.

## Figures and Tables

**Table 1:** Illumina sequencing metrics.

Sample	Reads	Q30 (%)	De-Hosted Reads
e-swab_fabric	36,368,136	94.5	6,485,544
e-swab_leather	25,721,944	96.85	3,072,349

**Table 2:** ONT sequencing metrics.

Sample	Reads	Mean Read Length (bp)	De-Hosted Reads
e-swab_fabric	100,667	9702.3	100,943
e-swab_leather	69,068	9996.3	67,459

**Table 3:** Taxonomic classification of leather chair samples, showing microbial presence and abundance across Illumina and ONT sequencing technologies.

Species	Category	Illumina—Leather		ONT—Leather	
		nt_rpm	nt Percent Identity	nt Count	nt Percent Identity
*Acinetobacter lwoffii*	bacteria	100.0147	98.69	15	94.18
*Aggregatibacter aphrophilus*	bacteria	942.1585	98.09	NA	NA
*Anoxybacillus caldiproteolyticus*	bacteria	23,597.2928	99.41	NA	NA
*Alphapolyomavirus quintihominis*	viruses	201.33268	99.22	16	93.48
*Betapapillomavirus*	viruses	865.60003	97.91	67	97.21
*Botrytis cinerea*	eukaryota	329.6903	99.41	NA	NA
*Corynebacterium* sp.	bacteria	NA	NA	125	95.66
*Clostridioides difficile*	bacteria	750.2735	99.55	NA	NA
*Cutibacterium acnes*	bacteria	1157.1739	99.83	NA	NA
*Desemzia incerta*	bacteria	14,401.4734	96.65	NA	NA
*Escherichia coli*	bacteria	966.2663	98.42	NA	NA
*Gammapapillomavirus 15*	viruses	169.4061	99.68	12	99.54
*Lactiplantibacillus plantarum*	bacteria	279.194289	96.83	41	94.82
*Leclercia adecarboxylata*	bacteria	675.3439	99.44	NA	NA
*Pseudomonas fluorescens* group	bacteria	399,454.705506	98.27	45896	96.42
*Rhodococcus globerulus*	bacteria	148.55612	99.66	NA	NA
*Staphylococcus* spp.	bacteria	69,663.3733	93.82	1649	92.79
*Staphylococcus haemolyticus*	bacteria	2843.0905	96.30	NA	NA
*Veillonella parvula*	bacteria	157.3522	96.30	NA	NA

NA = not detected.

**Table 4:** Taxonomic classification of fabric chair samples, showing microbial presence and abundance across Illumina and ONT sequencing technologies.

Species	Category	Illumina—Fabric		ONT—Fabric	
		nt_rpm	nt Percent Identity	nt Count	nt Percent Identity
*Achromobacter xylosoxidans*	bacteria	1322.43	99.39	NA	NA
*Delftia acidovorans*	bacteria	3136.41	98.1	NA	NA
*Escherichia coli*	bacteria	98,845.79	98.65	7493	99.38
*Ezakiella massiliensis*	bacteria	62.46	96.74	10	95.93
*Pseudomonas fluorescens* group	bacteria	51,943.45	94.07	8831	91.58
*Rhodococcus globerulus*	bacteria	377.57	99.07	NA	NA
*Staphylococcus* spp.	bacteria	1319.15	94.91	239	95.58

NA = not detected.

## Data Availability

NGS raw data have been uploaded in Zenodo open repository (https://zenodo.org/) and are available upon request at the following link https://doi.org/10.5281/zenodo.20086025.
